# Vaginal microbiota transplantation is a truly opulent and promising edge: fully grasp its potential

**DOI:** 10.3389/fcimb.2024.1280636

**Published:** 2024-03-22

**Authors:** Yiming Meng, Jing Sun, Guirong Zhang

**Affiliations:** ^1^ Department of Central Laboratory, Cancer Hospital of Dalian University of Technology, Liaoning Cancer Hospital and Institute, Shenyang, China; ^2^ Department of Biobank, Cancer Hospital of Dalian University of Technology, Liaoning Cancer Hospital and Institute, Shenyang, China

**Keywords:** vaginal microbiota transplantation, gynecological ailments, lactobacillus, synthetic bacterial consortia transplantation, application

## Abstract

Vaginal microbiota transplantation (VMT) is a cutting-edge treatment modality that has the potential to revolutionize the management of vaginal disorders. The human vagina is a complex and dynamic ecosystem home to a diverse community of microorganisms. These microorganisms play a crucial role in maintaining the health and well-being of the female reproductive system. However, when the balance of this ecosystem is disrupted, it can lead to the development of various vaginal disorders. Conventional treatments, such as antibiotics and antifungal medications, can temporarily relieve the symptoms of vaginal disorders. However, they often fail to address the underlying cause of the problem, which is the disruption of the vaginal microbiota. In recent years, VMT has emerged as a promising therapeutic approach that aims to restore the balance of the vaginal ecosystem. Several studies have demonstrated the safety and efficacy of VMT in treating bacterial vaginosis, recurrent yeast infections, and other vaginal conditions. The procedure has also shown promising results in reducing the risk of sexually transmitted infections and preterm birth in pregnant women. However, more research is needed to establish optimal donor selection, preparation, and screening protocols, as well as long-term safety and efficacy. VMT offers a safe, effective, and minimally invasive treatment option for women with persistent vaginal problems. It could improve the quality of life for millions of women worldwide and become a standard treatment option shortly. With further research and development, it could potentially treat a wide range of other health problems beyond the scope of vaginal disorders.

## Introduction

The human organism is a highly functional entity in symbiosis with microorganisms, and the microbiota has a profoundly interconnected association with the host’s overall health. The vaginal microbiota is a microbial community inhabiting the female vaginal mucosa and engaging in antagonistic, symbiotic, and primitive interactions, creating a complex micro-ecosystem within the human body. The fluctuations in vaginal microbiota can substantially impact both the physiological and immune functions of the vagina, ultimately influencing women’s reproductive health. This can lead to the development of various ailments, including vaginitis, cervical cancer, ovarian cancer, and other related diseases (Greenbaum et al., 2019; Mahajan et al., 2022).

The vaginal microbiota exhibits significant associations with the well-being of women and neonates. The composition of the vaginal microbiota in healthy women is characterized by a relatively uncomplicated structure, with *Lactobacillus* being the most significant and prevailing microorganism. *Lactobacillus* can attach to the receptors on the vaginal epithelial cells and colonize the surface of these cells, thereby creating a protective barrier. Bacteria synthesize polysaccharides and cell wall peptidoglycans to generate a biofilm on their surface, which protects against pathogenic microorganisms’ attachment and infiltration (Chee et al., 2020). Simultaneously, diverse metabolites produced by Lactobacillus, including lactic acid, H_2_O_2_, bacteriocin, and biological surface-active substances, exhibit inhibitory effects on the proliferation of vaginal pathogenic bacteria, promoting self-cleansing and enhancing vaginal health (France et al., 2022). *Lactobacillus* has been observed to elicit stimulation of the immune cells within the body, producing a diverse range of cytokines. It has also been noted to bolster local vaginal immunity, augmenting the mucous membrane’s anti-infective properties, and preserving the host’s reproductive capacity, thereby contributing to overall body health maintenance (Torcia, 2019).

The vaginal microenvironment typically maintains a state of equilibrium and stability through a complex interplay of antagonistic and symbiotic interactions. This delicate balance serves as the primary mechanism of protection against external infections. When the equilibrium of the stable state is disrupted, and the prevailing bacteria are substituted by either a resident flora or pathogenic bacteria that infiltrate the body, it can result in various bodily discomforts and elicit an inflammatory reaction (Laniewski et al., 2020; Graham et al., 2021). Given the intimate association between the microbiota and the onset of gynecological disorders, the significant prevalence and inadequate remission rate of such conditions pose a considerable detriment to female health (Balle et al., 2020). Thus, preserving a healthy micro-ecological environment in the vaginal region is crucial to preventing gynecological ailments. The physiological environment of the gastrointestinal tract and the female reproductive system share similarities, which may contribute to the pathogenesis of enteric and vaginal infections resulting from the proliferation of pathogens (Younes et al., 2018). Fecal microbiota transplantation (FMT) has recently emerged as a dependable and efficacious targeted therapy for gastrointestinal disorders (Meng et al., 2023). Is it achievable to perform vaginal microbiota transplantation (VMT) to directly transfer the entire vaginal microbiota of healthy women to patients, restore the balance of the vaginal microbiota in patients, and enhance their overall health? We intend to comprehensively explore the function of VMT in treating illnesses. This entails comprehending the processes by which VMT functions, its potential positive impacts, and its use in the medical sector. We will investigate how this novel process can be used as a therapeutic approach to addressing different ailments, highlighting its importance and potential impacts on health and disease management.

## The identification of VMT

**Figure 1:**
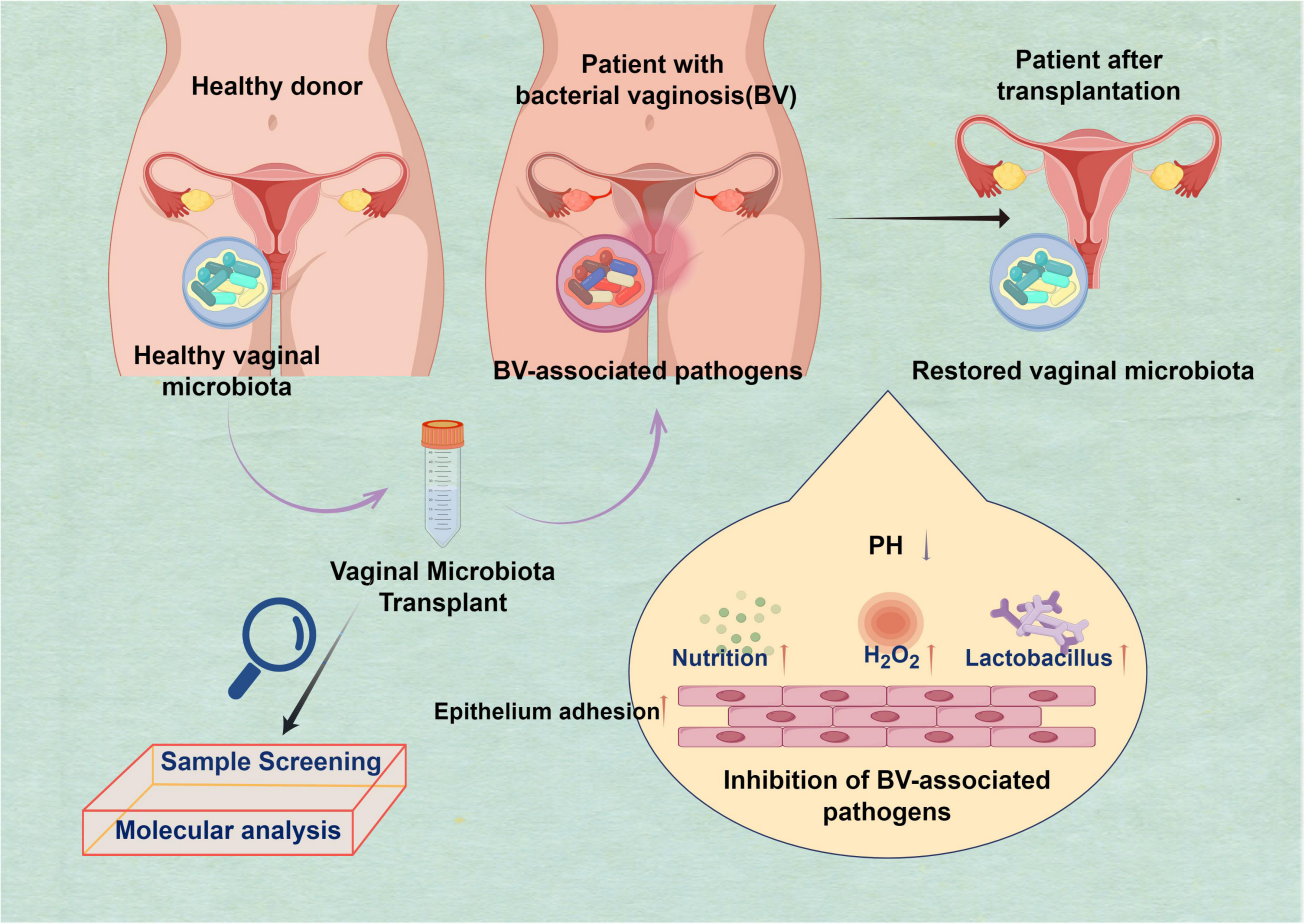
Harnessing beneficial vaginal microbiota transplantation (VMT) to Restore vaginal microbiota and combat bacterial vaginosis (BV). The purpose of the transplantation is to restore the harmonious equilibrium of the vaginal microbiota, which is often disturbed in cases of BV. This method presents a hopeful pathway for addressing this prevalent ailment by harnessing the potency of advantageous microorganisms to fight the detrimental ones: H_2_O_2_ has been enhanced; competition for nutrition has increased. Additionally, there has been an improvement in the capacity of epithelial cells to adhere, and the presence of Lactobacillus is abundant. The pH level is low, creating a functional and protective barrier.

In 1955, Dr. Herman L. Gardner conducted a study that intentionally induced bacterial vaginosis (BV) in a group of healthy older women by directly inoculating their vaginal microbiota with *Gardnerella vaginalis* (*G. vaginalis*)-positive samples obtained from other women (Bautista et al., 2016). The BV is prevalent among women worldwide and may result in severe health issues if left untreated, such as heightened vulnerability to other sexually transmitted infections and problems during pregnancy. Moreover, the microorganisms responsible for this illness may be transmitted via sexual intercourse, emphasizing a public health issue (Abou Chacra et al., 2021; Koyama et al., 2023). BV was observed to occur in 73% of the healthy female volunteers, specifically in 11 out of 15 individuals. In contrast, the introduction of pure *G. vaginalis* cultures into women’s bodies resulted in the development of BV in only one out of thirteen women. The present study serves as the foundation for the ongoing investigation into the efficacy of VMT as a prospective treatment for recurrent and intricate vaginal infections (**Figure 1**). Currently, there is ongoing research into two broad methodologies. A potential method for addressing BV involves the direct transplantation or inoculation of vaginal fluid obtained from a healthy individual into the vaginal tract of an individual experiencing BV—the second approach directly transplants particularly cultured derivatives into the vaginal region of individuals diagnosed with BV (Vieira-Baptista et al., 2022).

## The utilization of VMT

The bacterial diversity in the vaginal microbiota is comparatively lower than that found in the intestinal tract. The vaginal environment is primarily regulated by a diverse range of lactic acid bacteria to maintain homeostasis (Brown et al., 2023). However, the vaginal environment in an individual’s life is dynamic and presents complex circumstances. The composition of the vaginal microbiota is subject to dynamic changes over time, with fluctuations occurring not only during pregnancy and menopause but also on a shorter timescale ranging from days to months (Lewis et al., 2017; Tomas et al., 2020).

## Techniques for BV diagnosis

Recent progress in identifying BV techniques has resulted in various diagnostic tools. A traditional way of finding it has been to use a set of clinical signs called Amsel criteria (Holm et al., 2023). These include abnormal vaginal discharge, high vaginal pH, clue cells in a saline wet mount, and a fishy odor when potassium hydroxide is added to the vaginal fluid (Mohammadzadeh et al., 2014). However, the accuracy of these traditional methods can be variable, and they may only sometimes provide a definitive diagnosis (Abou Chacra et al., 2024). This has led to the development of more advanced methods that aim to overcome these limitations. These include molecular-based techniques that detect specific bacterial genes or species associated with the condition (Savicheva, 2023). For instance, polymerase chain reaction (PCR) methods have been developed to detect and quantify the specific bacterial species associated with BV (Bretelle et al., 2023). These techniques are susceptible and specific, providing a more accurate diagnosis than traditional methods. Another approach is the use of next-generation sequencing (NGS) technologies to analyze the entire vaginal microbiome (Savicheva et al., 2023). This approach can provide a more comprehensive picture of the bacterial species present and their relative abundances, which can help diagnose BV. Despite progress, the subject of BV identification is continually developing, with current research aimed at enhancing the precision, efficiency, and cost-efficiency of these approaches (Redelinghuys et al., 2020). Advancements in understanding the vaginal microbiota and its connection to BV are expected to lead to the development of more precise and effective identification tools in the future.

## VMT in bacterial vaginosis

The primary etiology of BV is the dysbiosis of the vaginal microbiota and its potential associations (Joseph et al., 2021). Vaginal dysbiosis may be caused by several circumstances, including the administration of antibiotics, which can eliminate beneficial bacteria; hormonal fluctuations like those seen during the menstrual cycle, pregnancy, or menopause; and lifestyle variables such as dietary choices and cleanliness habits. Furthermore, it is crucial to acknowledge that sexually transmitted illnesses and disorders may also cause dysbiosis (Han et al., 2021; Swidsinski et al., 2023). The female vaginal microbiota comprises diverse microorganisms that establish a stable ecological equilibrium, and perturbations in the vaginal milieu can disrupt this balance, leading to dysbiosis. From a clinical perspective, the primary approach to managing the condition involves the administration of antibiotics and other pharmacological agents to alleviate symptoms. However, this approach is limited to providing symptomatic relief and is associated with a high recurrence rate (Van De Wijgert and Verwijs, 2020; Chen et al., 2021b). A research team administered antibiotics to five patients with intractable BV to suppress their vaginal microbiota (Lev-Sagie et al., 2019). After a week had passed, the researchers obtained vaginal secretions from three female participants who had not experienced BV within the previous five-year period. Subsequently, a cervical injection was administered to each participant using fluid obtained from a solitary donor. Later, the investigators conducted regular follow-up assessments of the participants, monitoring their symptoms and analyzing their vaginal secretions. In the event of a recurrence of BV symptoms during the follow-up period, the treatment above may be repeated for a maximum of three instances. Out of the total of five patients, a majority of four individuals did not exhibit any signs of relapse symptoms during the 21 months after their most recent transplant. One patient showed incomplete remission in both clinical and laboratory characteristics. None of the five women exhibited any adverse effects. Notably, remission was observed in three patients who required repeated VMT, with one patient undergoing a donor change to achieve a sustained clinical response. Further investigation is necessary to determine the therapeutic effectiveness of VMT in women who suffer from persistent and recurring BV. This should be accomplished through randomized clinical trials incorporating a placebo control group.

The present investigation involved isolating and screening probiotic strains from the vaginal secretions of healthy women with inhibitory effects against *G. vaginalis* (Li et al., 2023). The screened strains were composed of synthetic microbiota. *In vivo*, verification of the efficacy of VMT was conducted, and a comprehensive investigation was carried out to elucidate the mechanism by which synthetic microbiota transplantation ameliorates BV. The present study involved the isolation and purification of lactic acid bacteria from the vaginal secretions of five healthy women, which exhibited the ability to inhibit the growth of *Gardnerella*. These strains hold potential as promising candidates for the treatment of BV. Subsequently, a model of BV was established to investigate the impact of VMT, comprising *Lactobacillus crispatus* (*L. crispatus*), *Lactobacillus plantarum*, *Lactobacillus salivarius*, and *Lactobacillus rhamnosus*, on the imbalance of vaginal microbiota. The experiment’s findings indicate that applying Synthetic Bacterial Consortia Transplantation (SBCT) and VMT treatment reduced the bacterial load of *G. vaginalis* in the vaginal region of mice afflicted with BV. Furthermore, the present investigation examined the impacts of the transplantation of synthetic microbiota and vaginal microbiota on the microbiota’s diversity and structure. Additionally, the potential correlation between alterations in the microbiota and their predictive capacities was explored. The results of the α-diversity and ß-diversity analyses indicate that the microbial diversity of the microbiota exhibited a degree of recovery after treatment and that the structure of the microbiota became more stable and similar. The objective is to reinstate the disrupted vaginal microbiota to a state of equilibrium characterized by elevated levels of lactic acid bacteria and reduced levels of *Escherichia*, thereby preventing the recurrence of vaginal microbiota imbalance. The utilization of the cluster of homologous genes (COG) approach in the present study revealed that the synergistic action of the microbiota facilitates the active gene expression observed in samples associated with BV. In brief, synthetic and VMT have effectively modulated innate and adaptive immune responses in mice afflicted with BV. Additionally, this approach promotes the growth of lactic acid bacteria while inhibiting the growth of pathogens, thereby maintaining the balance of vaginal microbiota.

## The protection of uterine health

Research has substantiated that the transfer of microbial communities between various locations within the female reproductive system is a crucial determinant of the well-being of the uterine cavity (Wang et al., 2021). The authors initially conducted profiling of the microbial samples obtained from the vaginal and uterine regions of 145 female subjects. This was supplemented with an in-depth analysis of publicly available data and animal experiments to gain insights into the phenomenon of microbial translocation within the female reproductive tract and its potential impact on uterine health. The researchers also discovered that the transplantation of specific strains of vaginal microbiota into the vaginal cavity of rats elicits either a decrease or an increase in symptoms similar to endometritis. The research revealed that *Lactobacillus* exerts a protective influence on endometrial health. This was evidenced by the significant reduction in inflammatory markers in rats that underwent vaginal transplantation of *Clostridium* and *Lactobacillus*, compared to those that received only *Clostridium*. The findings were corroborated through microbiota sequencing. A significant amount of *Lactobacillus* in the vaginal region can potentially exert a cross-site effect, leading to a decrease in the prevalence of detrimental bacteria within the uterine cavity, thereby promoting healthy microbiota within the uterine cavity. This research elucidated, for the first time, the attributes of the interplay and co-variation between the uterine cavity and vaginal microbiota. It was discovered that the vaginal microbiota could serve as an indicator of the health condition of the uterine cavity or aid in the prompt identification, prevention, and treatment of endometrial ailments. The present investigation established a theoretical framework for implementing VMT as a clinical intervention for intrauterine pathologies, enhancing endometrial receptivity and preserving reproductive well-being. Surprisingly, research revealed that the vaginal probiotic *L. crispatus* has a significant impact on sperm activity and may potentially decrease the occurrence of pregnancies due to its ability to adhere, which might explain some cases of infertility that are now unexplained (Li et al., 2021). Hence, it is advisable to exercise more care while using *L. crispatus* as a vaginal probiotic in women of reproductive age, particularly in cases where the female partners have male partners with defective sperm. They posited a novel approach to safeguarding the well-being of the uterine cavity through manipulating vaginal microecology.

## Treatment with antibiotics and VMT slows the development of endometrial disease

Endometrial disease refers to many disorders that impact the endometrium, which is the inner lining of the uterus. Endometriosis and endometritis are prevalent and significant disorders (Heil et al., 2023; Shuai et al., 2023). Endometritis is inflammation or irritation of the endometrium. An infection, such as chlamydia, gonorrhea, tuberculosis, or a combination of normal vaginal bacteria, often triggers the condition (Davis et al., 2023). Research conducted on rat models of vaginal dysbiosis has shown that VMT may be a beneficial treatment in lowering inflammation, promoting the growth of *Lactobacilli*, and alleviating symptoms similar to endometritis (Chen et al., 2021a; Lu et al., 2022). Endometriosis (EMS) is a medical condition typified by symptoms that are both chronic and inflammatory and are dependent on estrogen. These symptoms can be pretty bothersome to those who suffer from the disease (Chapron et al., 2019). The determination of EMS diagnosis primarily relies on pathological analysis after surgical excision, whereas the etiology of EMS remains inadequately understood. Surgical excision and pharmacotherapy, encompassing analgesics and hormone manipulation, particularly administering gonadotropin-releasing hormone analogs (GnRH-a), are frequently employed modalities (Gao et al., 2022). However, they are associated with considerable costs and adverse effects. Limited research has been conducted on the vaginal microbiota of individuals diagnosed with endometriosis. The EMS model was established in female mice through the intraperitoneal administration of fragments obtained from donor mice, as evidenced by a research study (Lu et al., 2022). Subsequently, the mice were administered a combination of antibiotics (administered intravaginally) and inhibitors targeting the NF-κB signaling pathway (delivered via intraperitoneal injection). The findings indicate that the ectopic lesions were suppressed. Furthermore, a noteworthy reduction was observed in the levels of inflammatory cytokines such as IL-1β, IL-6, and TNF-α in the peritoneal fluid, as well as in the expression of the cell proliferation marker KI-67 and macrophage marker Ionized calcium-binding adaptor molecule 1 (IBA1) in ectopic lesions, when compared to the corresponding values in the control group. Similar outcomes were noted through the implementation of VMT and subcutaneous administration of leuprorelin acetate (LA), a form of GnRH-a, in mice exhibiting symptoms of endometriosis. This study’s findings indicate that administering antibiotics or VMT is efficacious in treating endometriosis in murine models. Nonetheless, the dissimilarity between the vaginal microbiota of humans and mice necessitates further investigation into its mechanism and potential clinical applications.

## Is the practice of “Bacterial Baptism” a viable solution for the restoration of lost microbiota in infants born via Cesarean section?

Establishing maternal microbiota in neonates is crucial to their growth and well-being (Bhattacharyya et al., 2023). A scientific research team comprising multiple centers in the United States has discovered that neonates delivered via cesarean section can partially restore their intestinal microbiota by being swabbed with maternal vaginal microbes immediately after birth (Dominguez-Bello et al., 2016). The study participants consisted of 18 mothers, seven having undergone vaginal delivery and 11 having undergone cesarean delivery. Four infants born via cesarean delivery underwent a vaginal microbial scrubbing procedure. The investigators initially inserted sterile moist gauze into the vaginal canal of the pregnant participant and allowed it to remain for 60 minutes. Subsequently, the gauze was removed prior to the commencement of the cesarean delivery, placed in a sterile container, and stored at ambient temperature until the procedure was initiated. Immediately following delivery, it is recommended to utilize the gauze containing the maternal vaginal microbiota to cleanse the newborn’s lips, cheeks, chest, arms, legs, genitalia, and perianal region sequentially, concluding with the back. Upon completion of the scrubbing procedure, the entire process is terminated. The efficacy of the scrub was demonstrated in a sample of four infants during testing. Over 30 consecutive days, the microbiota of infants who underwent the scrub test exhibited typical maternal vaginal microbiota. In contrast, the microbiota of infants who did not experience the scrub test displayed characteristics of environmental microbiota. Furthermore, the investigators intend to broaden the scope of the study by enlisting 1,200 participants and prolonging the subsequent inquiry for 3-5 years to investigate the effects of their therapeutic approaches on the prevalence of allergies and asthma among the examined infants. If the research proves successful, it has the potential to revolutionize the field of human reproduction and alter its historical trajectory.

Evidence suggests that the risk of allergies, asthma, obesity, and other diseases in offspring is higher when maternal microbial transmission is delayed or interrupted, as is the case with cesarean section and early antibiotic exposure. Numerous investigations currently examine the potential of transferring maternal vaginal microbiota to progeny (Jasarevic et al., 2018; Giordani et al., 2023; Hashiramoto et al., 2023). A recent study has revealed that establishing vaginal microbiota during delivery can be replicated by constructing a mouse model of cesarean delivery that mimics the human vaginal microbiota (Jasarevic et al., 2021). The present investigation discloses the enduring consequences of distinct maternal vaginal microbiota on progeny’s metabolic, immune, and cerebral development, and the interplay of maternal endogenous perturbations modulates this microbial influence. The study mentioned above furnishes a crucial theoretical framework for future investigations about the impact of human vaginal microbiota on the well-being of progeny and the transfer of maternal vaginal microbiota. This paper presents valuable insights despite the need for comprehensive validation and in-depth exploration of underlying mechanisms. The present paper presents mouse models and preliminary analysis outcomes of diverse human microbes, which serve as a foundation for future research on the intergenerational transmission effects of maternal vaginal microbiota, maternal gut microbiota, milk microbiota, and skin microbiota. The present article underscores maternal exposure to unfavorable environmental factors during prenatal development can impact the offspring’s response to bacterial colonization after birth. Clinical trials investigating the efficacy of maternal microbiota transplantation ought to gather data about adverse environmental exposures experienced by the mother during pregnancy and analyze the maternal microbiota’s composition and functionality. This statement suggests that assessing the viability and security of maternal microbiota transplantation is necessary to enhance the well-being of both expectant mothers and their offspring.

The research above indicates that the vaginal microbiota of the mother has a significant impact on the maturation and advancement of neonates, particularly in the establishment of gut microbiota. Nonetheless, research endeavors exist that employ random allocation to divide infants delivered via cesarean section into two groups: an intervention group that receives 3 ml of maternal vaginal microbiota solution orally and a control group that receives sterile water (Wilson et al., 2021). Infants delivered vaginally are utilized as the control group. The research compared the gut microbiota composition and associated biological processes in infants delivered via cesarean section at 1 and 3 months of age. The results indicated that there were no significant variations between the two cohorts. Infants delivered via cesarean section exhibited a decrease in the prevalence of certain bacteria and a reduction in the expression of specific biosynthetic pathways compared to infants delivered vaginally. None of the studies reported any significant unfavorable incidents associated with the intervention. The research did not reveal any advantageous outcomes of maternal vaginal microbiota being orally administered to neonates delivered through cesarean section. Additional research has indicated a negligible variance in the composition of *Lactobacillus* originating from the maternal vaginal canal in the intestinal microbiota of neonates delivered vaginally versus those produced via cesarean section (Shao et al., 2019). This raises doubts regarding the effectiveness of vaginal microbiota seeding.

## The potential application of VMT in tumor therapy is a subject of academic interest

**Figure 2:**
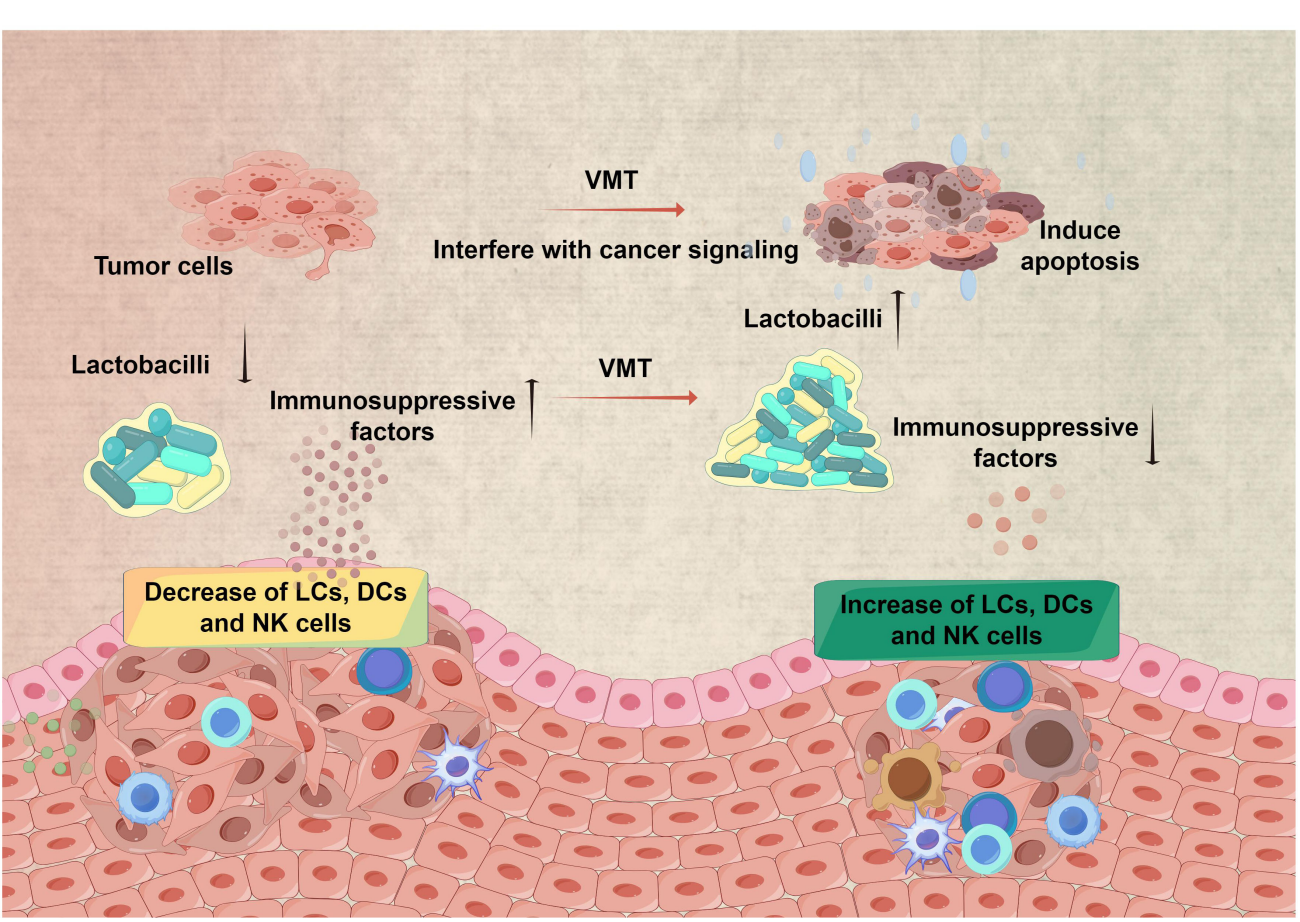
Restoring a healthy microbiota for improved therapy efficacy in cervical cancer. VMT has the potential to contribute to cervical cancer treatment via many channels. Restoring a healthy microbiota by VMT has the potential to improve the local immune response, leading to better elimination of HPV and lowering the likelihood of HPV persisting and progressing to cervical cancer. Furthermore, altering the composition of the vaginal microbiota has the potential to impact the effectiveness of therapy for cervical cancer. (All figures were drawn by FigDraw).

Cervical cancer is a prevalent malignancy associated with human papillomavirus infection (HPV) and ranks as the fourth most frequent cancer among women globally (Cohen et al., 2019). The cervix’s squamocolumnar junction, or the transformation zone, is highly vulnerable to HPV infection and is where cervical cancers originate (Bewley, 2022). In recent years, increasing evidence indicates that the vaginal microbiota may play a significant role in the development of cervical cancer (**Figure 2**) (Mitra et al., 2016; Kyrgiou and Moscicki, 2022). Studies in the field of epidemiology have demonstrated correlations between a variety of vaginal microbiota that *Lactobacillus* and the occurrence and continuation of HPV infection do not dominate (Kyrgiou et al., 2017). The study on 100 women revealed a correlation between precancerous lesions and cervical cancer, with decreased *Lactobacillus* dominance and increased diverse vaginal microbiota (Laniewski et al., 2018). About particular taxa, the predominant microbiota that exhibited enrichment in women infected with HPV or those with dysplasia or cancer is not restricted to microorganisms linked with BV, such as *Gardnerella*, *Atopobium*, *Prevotella*, *Megasphaera*, *Parvimonas*, *Peptostreptococcus*, *Anaerococcus*, *Sneathia*, *Shuttleworthia*, and *Gemella*. They also encompass those that give rise to other types of dysbiosis, including *Streptococcus agalactiae* and *Clostridium*. The potential application of VMT in the treatment of cervical cancer can be deduced from the observed interplay between HPV and vaginal microbiota.

The potential involvement of the microbiota in the development of endometrial cancer is suggested by the observed correlation between gut microbiota, estrogen metabolism, and obesity (Mitchell et al., 2015). Recent developments in sequencing technologies have revealed that estrogenic compounds can influence the microbial communities in the vaginal and distal regions. Furthermore, it has been observed that the estrobolome can modify the circulating estrogen levels (Riganelli et al., 2020; Barczynski et al., 2023). Given the direct impact of estrogen levels on the health and homeostasis of the vaginal microenvironment, estrogen may also influence the axis between the gut and vaginal microbiotas (Kwa et al., 2016). A study report indicates a significant correlation between the co-occurrence of *Atopobium* and *Porphyromonas*, an abnormal vaginal pH level exceeding 4.5, and the status of endometrial cancer (Walther-Antonio et al., 2016). To confirm the link between vaginal microbiota and endometrial cancer, however, more extensive cohort studies are required in the future.

Ovarian cancer is a highly lethal neoplasm affecting the female population (Armstrong et al., 2022). The development of ovarian tumors has been strongly associated with chronic infections with sexually transmitted pathogens and inflammation in the genital tract, much like the association observed with endometrial cancer (Schoutrop et al., 2022). According to a study, the prevalence of vaginal microbial community type, which exhibits less than 50% dominance of *Lactobacillus* spp., is significantly higher in ovarian cancer cases or cancer-related *BRCA1* mutations (Nene et al., 2019). The potential impact of microbial dysbiosis and differences in vaginal microbiota composition between healthy individuals and those with cancer should be considered as a contributing factor in ovarian cancer progression (Verstraelen, 2019). Research has indicated that vaginal dysbiosis and abnormal microbes play a role in the development and advancement of ovarian cancer and is frequently observed as a complication of anti-cancer treatment. Given the significant potential, it is imperative to conduct further investigation into the therapeutic benefits of modulating the vaginal microbiota and VMT in the context of treating gynecological cancers.

## Conclusion

VMT is a promising area of innovation within women’s health. As further investigation into the effectiveness of the procedure above persists, it could potentially be utilized in the management of additional ailments associated with the vaginal microbiota. VMT has garnered significant interest in contemporary times owing to its capacity to overcome conventional therapies’ constraints and reinstate microbiota’s natural equilibrium in the vaginal microbiota, thereby mitigating the likelihood of future infections. Notably, the procedure above is currently regarded as experimental, and its accessibility is limited. Individuals who express interest in the aforementioned medical procedure are advised to seek consultation with a healthcare professional to ascertain their eligibility and engage in a comprehensive discussion regarding the potential advantages and drawbacks of the system. Furthermore, as VMT entails transferring biological material from one person to another, ethical issues must be considered. These challenges include donor screening and selection, informed consent, privacy, and confidentiality. It is essential to adequately address ethical problems and establish strong ethical standards before the widespread use of VMT as a treatment technique. Ultimately, VMT has the potential to advance women’s health but is still in its early stages of development. It requires extensive investigation and inquiry. We are eager to see how VMT might be used to enhance preventative and treatment efforts for various health conditions associated with abnormalities in the vaginal microbiota as our knowledge of these intricate interactions progresses.

## Author contributions

YM: Funding acquisition, Writing – original draft, Writing – review & editing. JS: Resources, Writing – review & editing. GZ: Investigation, Writing – review & editing.
